# A Late Attempt to Involve End Users in the Design of Medication-Related Alerts: Survey Study

**DOI:** 10.2196/14855

**Published:** 2020-03-13

**Authors:** Melissa Therese Baysari, Wu Yi Zheng, Bethany Van Dort, Hannah Reid-Anderson, Mihaela Gronski, Eliza Kenny

**Affiliations:** 1 Faculty of Health Sciences The University of Sydney Sydney Australia; 2 Centre for Health Systems & Safety Research Australian Institute of Health Innovation Macquarie University Sydney Australia; 3 Macquarie University Hospital Sydney Australia

**Keywords:** alert fatigue, alerting, medication alert systems, clinical decision support, hospital information systems

## Abstract

**Background:**

When users of electronic medical records (EMRs) are presented with large numbers of irrelevant computerized alerts, they experience alert fatigue, begin to ignore alert information, and override alerts without processing or heeding alert recommendations. Anecdotally, doctors at our study site were dissatisfied with the medication-related alerts being generated, both in terms of volume being experienced and clinical relevance.

**Objective:**

This study aimed to involve end users in the redesign of medication-related alerts in a hospital EMR, 4 years post implementation.

**Methods:**

This work was undertaken at a private not-for-profit teaching hospital in Sydney, Australia. Since EMR implementation in 2015, the organization elected to implement all medication-related alert types available in the system for prescribers: allergy and intolerance alerts, therapeutic duplication alerts, pregnancy alerts, and drug-drug interaction alerts. The EMR included no medication administration alerts for nurses. To obtain feedback on current alerts and suggestions for redesign, a Web-based survey was distributed to all doctors and nurses at the site via hospital mailing lists.

**Results:**

Despite a general dissatisfaction with alerts, very few end users completed the survey. In total, only 3.37% (36/1066) of doctors and 14.5% (60/411) of nurses took part. Approximately 90% (30/33) of doctors who responded held the view that too many alerts were triggered in the EMR. Doctors suggested that most alerts be removed and that alerts be more specific and less sensitive. In contrast, 97% (58/60) of the nurse respondents indicated that they would like to receive medication administration alerts in the EMR. Most nurses indicated that they would like to receive all the alert types available at all severity levels.

**Conclusions:**

Attempting to engage with end users several years post implementation was challenging. Involving users so late in the implementation process may lead to clinicians viewing the provision of feedback to be futile. Seeking user feedback on usefulness, volume, and design of alerts is extremely valuable; however, we suggest this is undertaken early, preferably before system implementation.

## Introduction

Many studies have shown that medication-related computerized alerts embedded in hospital electronic medical records (EMRs) can result in significant changes to prescriber behavior [1]. For example, introduction of dosing alerts for psychotropic medications in a US tertiary hospital led to an increase in the prescription of recommended daily doses from 19% to 29% and a reduction in the incidence of tenfold dosing errors from 5% to 3% [2]. However, studies have also shown that computerized alerts can have no impact or a negative impact on prescribing [1]. One of the main factors hampering alert effectiveness is alert overload. When users are presented with large numbers of irrelevant alerts, they experience alert fatigue [3], begin to ignore alert information [4], and override alerts (ie, click past the alert window) without processing or heeding alert recommendations [5].

Despite international efforts to improve medication-related alerts, alert override rates remain as high as they were over a decade ago [6]. Alert fatigue appears to be a widespread problem for users of EMRs, but there is limited evidence available on what constitutes a *tolerable* volume of irrelevant alerts for prescribers [7,8]. How many is too many before alert fatigue sets in? With no answer to this question, many hospitals have chosen to enable the main decision support alerts available in their EMR, including drug-drug interaction, allergy, and dose range alerts, with some also implementing pregnancy and therapeutic duplication warnings [9]. Our site was one such hospital, implementing all available medication-related alert types for prescribers.

In this study, we describe an attempt that was made to involve end users in the redesign of medication-related alerts several years post implementation. Anecdotally, via informal discussions with doctors, we learned that doctors were dissatisfied with the alerts being generated, both in terms of volume being experienced and clinical relevance. We administered a Web-based survey to doctors and nurses to more systematically capture their views of medication-related alerts embedded in the hospital EMR and their suggestions for redesign.

## Methods

### Context

This work was undertaken at a private not-for-profit teaching hospital, with approximately 150 beds, in Sydney, Australia. In 2015, the hospital implemented a single EMR in all wards and areas of the hospital. Before EMR implementation, a suite of fragmented clinical information systems was in place (an intensive care unit system, a general ward system, a medication management system, and a patient administration system). The single EMR was introduced to better support integration of care and to ensure efficient and effective delivery of health services. With respect to medication management, the system allowed prescribing, pharmacy review, and medication administration.

### Implementation of Medication-Related Alerts

**Figure 1:**
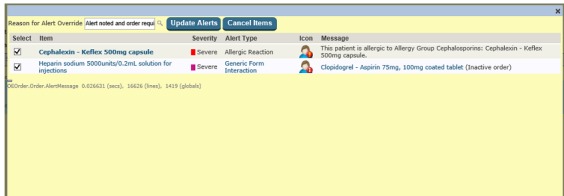
An example showing two alerts that have been triggered simultaneously, an allergy alert and a drug-drug interaction alert.

A core group of clinicians and administrative staff (information technology and billing) were consulted before EMR implementation, which included mainly heads of departments and units. Since EMR implementation, the organization elected to implement all medication-related alert types available in the system for prescribers: allergy and intolerance alerts, therapeutic duplication alerts, pregnancy alerts, and drug-drug interaction alerts. Not long after implementation, the alert configuration was changed so that only alerts classified as *severe* were displayed to doctors. All alerts were interruptive, appearing in an alert window that prevented prescribers from continuing with their orders until alerts were acknowledged. This could be done by selecting an override reason from a drop-down menu that appeared at the top of the alert screen (see Figure 1). The EMR included no medication administration alerts for nurses.

### User Engagement: Web-Based Survey

A Web-based survey was distributed to all doctors (n=1066) and nurses (n=411) at the site via hospital mailing lists (see Multimedia Appendix 1). Participation in the survey was voluntary, and no reimbursement was provided for participation. The survey was codeveloped with key stakeholders (researchers, doctors, nurses, pharmacists, and implementation staff) and comprised some basic demographic questions, questions on the current alerts in the system (for prescribers), and questions on preferences for changes to the system (for prescribers and nurses)—see Multimedia Appendices 2 and 3. Following distribution of the survey, a single reminder was sent out after 2 weeks. The survey was closed after 1 month.

### Data Analysis

Descriptive statistics were used for analysis of quantitative data. A general inductive approach was used for analysis of qualitative responses. Responses to free-text questions were reviewed independently by 2 researchers (WZ and BD) who came together to reach a consensus on key themes related to current alerts and redesign recommendations.

Ethics approval was obtained from the hospital’s human research ethics committee (reference number 5201833414318).

## Results

### Survey Respondents and Response Rate

In total, 36 doctors and 60 nurses responded to the survey, representing a response rate of 3.37% (36/1066) and 14.5% (60/411), respectively (see Table 1).

**Table 1:** Characteristics of 36 doctors and 60 nurses that responded to the survey. (Not all participants responded to all questions).

Respondents			Respondents who responded to the question, n (%)
**Prescribers**			
	**Roles**		
		Accredited practitioner—anesthetics	23 (64)
		Accredited practitioner—medical	4 (11)
		Accredited practitioner—surgical	6 (17)
		Registrar	1 (3)
		Intern	1 (3)
	**Days per week working at hospital**		
		Less than 2	23 (64)
		2-3	7 (19)
		4-5	6 (17)
	**Setting of EMR^a^ use**		
		Inpatient	36 (100)
		Outpatient	2 (5)
	**Frequency with which they prescribe in EMR**		
		Multiple times a day	27 (75)
		Once in the day	2 (6)
		Only on some days	2 (6)
		Rarely	4 (11)
		Never	1 (3)
	**Experience using other EMR systems**		
		Yes	18 (55)
		No	15 (45)
**Nurses**			
	**Roles**		
		Registered nurse	57 (95)
		Endorsed enrolled nurse	3 (5)
	**Days per week working at hospital**		
		1-2	5 (8)
		3-4	23 (38)
		5-6	32 (53)
	**Experience using other EMR systems**		
		Yes	24 (41)
		No	35 (59)

^a^EMR: electronic medical record.

Some respondents indicated that they were pleased the evaluation was being done:

Thank you for doing something to fix this. Please involve clinicians.

#02

However, on the whole, most doctors appeared highly dissatisfied with the EMR in general and made sarcastic or sweeping comments about the system in the free-text boxes of the survey. For example, a doctor said:

The whole system is user unfriendly, please do not give it another band-aid.

#21

### Findings Related to the Volume of Alerts Being Triggered in the Electronic Medical Record for Doctors

As shown in Table 2, approximately 90% (30/33) of doctors who responded held the view that too many alerts were triggered in the EMR, and only 9% (3/33) doctors indicated that the number of alerts was *right*.

When asked to indicate how frequently they read the alerts, responses were mixed, as shown in Table 3.

The free-text responses received in response to the question “I only read the alerts in the EMR when...” reflected this variability. Examples from doctors included:

Severe alert fatigue—I now disregard all XX (EMR) alerts and check my own prescribing as I would have done when medication charts were on paper.

#16

I scan them very quickly, but don’t read them fully. It’s almost impossible to prescribe in XX (the EMR) without creating alerts—so they don’t really “alert” me.

#13

I always read them.

#11

**Table 2:** Doctors’ views on the current alert burden in the electronic medical record (N=33 responses).

Response option	Frequency of doctors who responded to the question, n (%)
Far too many alerts, most need to be removed	19 (58)
Too many alerts, some need to be removed	11 (33)
About the right number of alerts	3 (9)
Too few alerts, some need to be added	0 (0)

**Table 3:** Frequency with which doctors reported that they read alerts in the electronic medical record.

Response option	Frequency of doctors who responded to the question, n (%)
Never	4 (12)
Rarely	6 (18)
Sometimes	10 (30)
Often	4 (12)
Always	9 (27)

### Findings Related to the Usefulness of Each Alert Type for Doctors

When asked to rate each alert type on a Likert scale of usefulness, allergy and intolerances alerts were rated most positively. As shown in Table 4, these alerts were viewed to be *sometimes useful* by approximately half of the doctors in the survey. The other alert types were rated as *never* or *rarely useful* by the majority of doctors. When asked to indicate which alert type was the most useful, 60% (20/33) doctors selected allergy and intolerances alerts, and 24% (8/33) indicated that none of the alerts were useful.

Interestingly, when asked which of the alert types they would remove from the EMR (with more than one option possible), approximately half of the doctors (17/36, 47%) indicated that they would remove therapeutic duplication alerts, pregnancy alerts, and drug interaction alerts. In addition, 19% (7/36) also indicated that they would remove allergy and intolerance alerts.

**Table 4:** Doctors’ views on alert usefulness.

Response option	Frequency, n			
	Allergy and intolerances alert	Therapeutic duplication alert	Pregnancy alert	Generic drug interaction alert
Never useful	8	11	12	12
Rarely useful	5	13	15	12
Sometimes useful	17	7	6	10
Often useful	6	5	3	2

### Doctors’ and Nurses’ Preferences for Changes to the Alerting System

Nurses did not receive medication administration alerts in the EMR; however, 97% (58/60) of the nurse respondents indicated that they should. When asked what types of alerts nurses should receive, most nurses selected all the alert types available to the doctors. Other suggested alert types included alerts for stat orders, overdue medications, blood thinning medications, dose alerts, and alerts warning when a nursing intervention was required (eg, digoxin).

When asked to indicate what alert severity level or levels should be included in the EMR, nurses and doctors expressed very different views. As shown in Table 5, nurses were more open to receiving alerts of all severities.

**Table 5:** Doctors’ and nurses’ preferences for alert severity.

Response option	Frequency of doctors who responded to the question, n (%)	Frequency of nurses who responded to the question, n (%)
Only severe alerts	12 (36)	1 (2)
Severe and moderate alerts	20 (61)	19 (33)
All alerts, including minor alerts	1 (3)	38 (66)

 In response to a request for suggested changes to the alerts in the EMR, the most frequent response from doctors was to reduce alert numbers primarily by making the alerts more relevant. For example, doctors said:

Too many for too many trivial issues.

#41

Real ones need to be more prominent. All the silly ones need to go.

#02

Many of the alerts are theoretical and are ignored in our everyday practice.

#17

Hard to tell which ones are useful and which ones are not...You are more likely to miss important alerts if you are bombarded with too many insignificant alerts.

#24

Other common suggestions from doctor respondents were related to alert design. Current alerts were described as *not user friendly,* and doctors suggested using color coding to highlight the type or severity of alerts and suggested making alert content more concise:

Perhaps they could be color coded e.g. the severe ones and e.g. multiple opioid prescribing alert be large and red, moderate orange, minor orange and smaller. The blue color is very neutral.

#20

This was consistent with what nurses viewed to be characteristic of a well-designed alert:

Brief but adequate information, highlighted (font and color).

#11

As simple as it can be.

#28

Some nurses also described what they believed to be the ideal content to be included in an alert screen. For example:

Alert specifies the issue clearly. Specifies the action required. The alert can be dismissed easily where appropriate. Contains the problem: e.g. duplicate drug.

#5

An alert that states: “ALERT,” not wordy but precise description of alert. An alert that is easy to understand.

#41

## Discussion

### Summary of Key Findings From the Survey

With respect to the current alerts in the EMR, prescribers reported that too many alerts were being triggered and that most alerts were not clinically relevant. As a consequence, alerts were not always being read, and many doctors reported experiencing alert fatigue. These findings confirm what had been suspected by the organization and are in line with many studies exploring user views of alerts [10,11]. Allergy alerts were perceived to be the most useful among the alert types, a finding also consistent with previous research [4,12]. With respect to recommended changes to the alerts, prescribers suggested that most alerts be removed and that alerts be more specific and less sensitive. Prescribers also recommended that alert interfaces be redesigned so that alert text is more concise, and color coding is used to signal alert type and severity of alerts. These suggestions were consistent with nurses’ preferences for alert content and display and reflect good *human factors* design [13,14].

### Lessons Learned From Our Attempt to Engage Users

Our survey highlighted some important lessons for the site and more broadly for other hospitals with plans to implement medication-related alerts in their EMR. Seeking user feedback on usefulness, volume, and design of alerts, even with this small-scale survey, proved to be extremely valuable; however, we suggest consulting users early and regularly. Our low response rate could in part reflect staff not checking emails or not having enough time to action emails. However, based on the free-text responses we received, it more likely reflected a workforce feeling disengaged and dissatisfied with the alerting system and associated implementation process. Early consultation would have allowed user views to be captured before negative mindsets were established and may have minimized any perception that the provision of feedback was futile. Continuous consultation with users is needed to ensure alert volume remains manageable and alert content remains relevant.

We recommend that organizations involve end users in the decision-making processes surrounding alert selection before EMR rollout as well as in implementation and ongoing evaluation. Involving users in EMR implementation more broadly has been shown to facilitate a sense of ownership of the system among users and to result in an EMR that aligns well with the needs of end users rather than the needs of the information technology department or leadership staff [15]. Consistent with previous research [16], our findings confirm that user engagement is particularly important for medication-related alerts. Engaging end users in the selection and implementation processes for alerts will also increase user awareness of the challenges associated with alert optimization (eg, vendor restrictions and complexity associated with identifying a set of *high priority* alerts).

One of the interesting findings that emerged from our survey was that in contrast to doctors, nurses showed a strong preference for enabling all alert types of all severities for nurses in the EMR. This could reflect their limited experience with alerts, as a common misconception held by clinicians who do not experience alerts themselves is *the more alerts, the better*. To allow end users to make informed decisions about alert selection, we recommend providing current users and prospective users with data on alert rates (eg, 50% of your orders/administrations/reviews will trigger an alert) to facilitate an understanding of the potential impact of alerts on their work and educating staff on the well-known risks associated with alert overload.

### Conclusions

Our attempt to engage users in the redesign of medication-related alerts in an EMR was largely unsuccessful, with only a small number of doctors and nurses partaking in our survey. This likely reflects the delay between implementation and seeking feedback from end users. However, the feedback we received from survey respondents on usefulness, volume, and design of alerts proved to be extremely valuable. We recommend hospitals adopt a *less-is-more* approach and work closely with all end user groups before EMR implementation to determine the types, design, and content of alerts and implement these in their systems.

## Appendix

Multimedia Appendix 1 Recruitment emails.

Multimedia Appendix 2 Doctor survey.

Multimedia Appendix 3 Nurse survey.

## Abbreviations

EMR: electronic medical record
