# Supplementary material for: A Late Attempt to Involve End Users in the Design of Medication-Related Alerts: Survey Study
Source: J Med Internet Res. 2020 Mar 13;22(3):e14855. doi: 10.2196/14855 (PMC7101499; doi:10.2196/14855)
Supplement: Multimedia Appendix 1 [file jmir_v22i3e14855_app1.docx]

**Appendix 1. Recruitment emails**

Dear Doctors,

We are looking for doctors to complete a short survey to help us identify which computerised alerts to keep in and take out of XXX (the EMR).

This research study is being undertaken by researchers from the Australian Institute of Health Innovation (AIHI), Macquarie University in collaboration with XXX (hospital name).

The study involves completing a 5-minute survey. Your survey responses are confidential and will be collated for analysis and reporting.

This study is completely voluntary and there are no consequences for not taking part. If you would like to participate, please click on the link below to complete the survey:

XXX

Before you begin, please see the attachment for a reminder of what each type of XXX (EMR) alert looks like.

Please do not hesitate to contact me if you require any more information.

Regards,

A/Prof Melissa Baysari

Dear Nurses,

We are looking for nurses to complete a short survey to help us identify which computerised alerts to keep in and take out of XXX (the EMR)..

This research study is being undertaken by researchers from the Australian Institute of Health Innovation (AIHI), Macquarie University in collaboration with XXX (hospital name).

The study involves completing a 5-minute survey. Your survey responses are confidential and will be collated for analysis and reporting.

This study is completely voluntary and there are no consequences for not taking part. If you would like to participate, please click on the link below to complete the survey:

XXX

Please do not hesitate to contact me if you require any more information.

Regards,

A/Prof Melissa Baysari
