# Supplementary material for: A Late Attempt to Involve End Users in the Design of Medication-Related Alerts: Survey Study
Source: J Med Internet Res. 2020 Mar 13;22(3):e14855. doi: 10.2196/14855 (PMC7101499; doi:10.2196/14855)
Supplement: Multimedia Appendix 2 [file jmir_v22i3e14855_app2.docx]

# Identification of alert types to include and exclude from XXX

**Prescriber survey**

Thank you for taking the time to participate in this survey on XXX alerts. Before you begin, remember that your responses are confidential and will be collated for analysis and reporting.

Please tick your role:

**Accredited practitioner – Anaesthetics ☐**

**Accredited practitioner – Medical ☐**

**Accredited practitioner – Surgical ☐**

**CMO ☐**

**Registrar ☐**

**Intern ☐**

In a typical week, how many days do you work at this hospital? _______

In what settings do you prescribe in XXX?

**Inpatient ☐ Outpatient ☐**

In a typical work day, how often would you prescribe in XXX?

**Multiple times in the day ☐**

**Once in the day ☐**

**Only on some days ☐**

**Rarely ☐**

**Never ☐**

The alerts currently active in XXX include allergy and intolerances alerts, therapeutic duplication alerts, pregnancy alerts, and generic drug interaction alerts. The attachment in your email will remind you what each alert type looks like.

1. How useful is each alert type in warning you about prescribing something potentially dangerous for your patients?

**Allergy and intolerances alert:** **Therapeutic duplication alert:**

Never useful **☐**  Never useful **☐**

Rarely useful **☐** Rarely useful **☐**

Sometimes useful **☐** Sometimes useful **☐**

Often useful **☐** Often useful **☐**

**Pregnancy alert:** **Generic drug interaction:**

Never useful **☐** Never useful **☐**

Rarely useful **☐** Rarely useful **☐**

Sometimes useful **☐** Sometimes useful **☐**

Often useful **☐** Often useful **☐**

1. What alert type in XXX is MOST USEFUL in warning you about prescribing something potentially dangerous for your patients?

**Allergy interaction alerts ☐**

**Pregnancy alerts ☐**

**Therapeutic duplication alerts ☐**

**Generic drug interaction alerts** **☐**

1. If you could remove one or more alert types from the current alert set in XXX, which types would you remove? (Tick all that apply)

**Allergy interaction alerts ☐**

**Pregnancy alerts ☐**

**Therapeutic duplication alerts ☐**

**Generic drug interaction alerts** **☐**

**None, I’d not remove any alert type** **☐**

1. How would you describe the current level of alerts in XXX?

**Far too many alerts, most need to be removed ☐**

**Too many alerts, some need to be removed ☐**

**About the right number of alerts ☐**

**Too few alerts, some need to be added ☐**

1. I read the alerts in XXX…

**Never ☐**

**Rarely ☐**

**Sometimes ☐**

**Often ☐**

**Always ☐**

1. I only read the alerts in XXX when… ________________________________________________________________________________________________________________________________________________________________________________________________________________________
2. What level of severity of alert do you think should be triggered in XXX?

**Only severe alerts, e.g. a potentially life-threatening drug-drug interaction ☐**

**Severe and moderate alerts, e.g. a therapeutic duplication where close monitoring is required (such as the prescription of multiple opioids) ☐**

**All alerts, including minor alerts ☐**

1. Are there any alerts that currently do NOT trigger in XXX that you think should be included?

________________________________________________________________________

________________________________________________________________________________________________________________________________________________________________________________________________________________________

1. Can you think of any changes needed to the alerts in XXX (e.g. they contain too much text)?

________________________________________________________________________

________________________________________________________________________________________________________________________________________________________________________________________________________________________

1. Do you have any other comments about XXX alerts?

________________________________________________________________________

________________________________________________________________________________________________________________________________________________________________________________________________________________________

1. Do you have any experience using other electronic medication management systems at other hospitals?

Yes **☐** No **☐**

1. If yes, can you think of any good things about the alerts used in those systems?

________________________________________________________________________

________________________________________________________________________________________________________________________________________________________________________________________________________________________
