# Supplementary material for: A Late Attempt to Involve End Users in the Design of Medication-Related Alerts: Survey Study
Source: J Med Internet Res. 2020 Mar 13;22(3):e14855. doi: 10.2196/14855 (PMC7101499; doi:10.2196/14855)
Supplement: Multimedia Appendix 3 [file jmir_v22i3e14855_app3.docx]

# Identification of alert types to include and exclude from XXX

**Nurse survey**

Thank you for taking the time to participate in this survey on XXX alerts. Before you begin, remember that your responses are confidential and will be collated for analysis and reporting.

Please tick your role:

**Registered Nurse ☐**

**Nurse practitioner ☐**

**Endorsed enrolled nurse ☐**

In a typical week, how many days do you work at this hospital? _______

Nurses currently **do not receive medication administration alerts in XXX**, but are able to view the alerts that have been triggered for prescribers.

1. Do you think nurses should receive medication administration alerts in XXX?

**Yes ☐ No ☐** (*Go to question 4*)

1. If yes, what alerts do you think should be included in XXX for nurses?

**Allergy alerts ☐**

**Pregnancy alerts ☐**

**Therapeutic duplication alerts ☐**

**Drug-drug interaction alerts** **☐**

**Other** __________________________________________________________________

________________________________________________________________________

1. What level of severity of alert do you think should be triggered in XXX for nurses?

**Only severe alerts, e.g. a potentially life-threatening drug-drug interaction ☐**

**Severe and moderate alerts, e.g. a therapeutic duplication where close monitoring is required (such as the administration of multiple opioids) ☐**

**All alerts, including minor alerts ☐**

1. In your opinion, what are the characteristics of a well-designed alert (e.g. contains concise information)?

________________________________________________________________________

________________________________________________________________________________________________________________________________________________________________________________________________________________________

1. Do you have any other comments about XXX alerts?

________________________________________________________________________

________________________________________________________________________________________________________________________________________________________________________________________________________________________

1. Do you have any experience using other electronic medication management systems at other hospitals?

**Yes ☐ No ☐**

1. If yes, can you think of any good things about the alerts used in those systems?

________________________________________________________________________

________________________________________________________________________________________________________________________________________________________________________________________________________________________
